# Annotations

**Published:** 1899-03-18

**Authors:** 


### ANNOTATIONS.

### Greek in Education.

A very distinguished member of the medical profession, notwithstanding that his energies have been turned into channels unconnected with its practice—namely, Sir George Birdwood—has furnished an introduction to a new edition of Charles Lamb's well-known adaptation of Chapman's translation of the "Odyssey," and has taken as his subject the educational influence of the Greek language and literature. His observations possess an especial opportuneness at a time when discussions about the character and quality of preliminary education for the medical profession are in the air, and deserve to be maturely considered by all whose position enables them to influence the reforms, in this direction, which are generally understood to be impending. It would hardly be an exaggeration of Sir George's views to say that he does not consider anyone who is not fairly acquainted with Greek to be educated at all; and his very forcible phraseology derives increased importance from the fact that the University of London has lately modified its matriculation examination in a contrary direction, by permitting candidates to take up "a branch of science" instead of Greek or a modern language. This course was accidentally taken without its being remembered that the examination, as thus altered, could no longer be accepted as a basis of registration for a medical student; and the University has consequently appealed to the Medical Council for a period of grace in this respect, in order that any students who have been misled by the regulations of the University, and may pass the matriculation in the present year, or in January, 1900, under the impression that the certificate will be sufficient, should be allowed to register as medical students, although they have not passed either in Greek or in any modern language. There could, of course, be no valid objection to granting such indulgence as a temporary measure, whatever may be thought of the principle involved. "Whenever I have been in a position," says Sir George Birdwood, "to direct the education of boys, after teaching them the alphabet—not beginning with it before their sixth year—and the English syllabary, I have forthwith put them through a course of heroical and romantic reading, beginning with 'Robin Hood' and 'Chevy Chase' and other national ballads, and then plunged them direct into the 'Iliad' and the 'Odyssey,' and finally the 'Æneid' of Virgil. When they have read each of these epics through thrice I have done with them. I have saved their souls alive. I know that they will do their duty in life, and wholeheartedly, never wasting a thought on its merely sordid rewards, for the 'Iliad' and the 'Odyssey' have made them masters of their fate." He says, again, that he has watched the careers of a large number of candidates for appointments in the civil and military services, successful and unsuccessful, and has found that those of them who happened to be well-grounded in Greek have invariably made their mark in after-life, there being nothing like Greek, not even the discipline of mathematics, for "drawing out" the faculties of the average human mind, raising it to the highest temper of which it is capable, sharpening it to its keenest edge, and polishing it to its utmost brightness, as a labouring weapon well prepared for its work in the world, whatever this may be. Similar views are well known to be entertained by many masters of English composition, who prize above all things the Greek and the French languages as instruments of education; and who deplore the learning of German, useful though it be as a medium of intercourse for tradespeople, but only too much calculated, by its tautology, its prolixity, and the involvement of its sentences, to render muddy the "pure well of English undefiled." We are much in sympathy with Sir George Birdwood's pronounced opinions, but we think his scheme of education has left out of account an instrument less fashionable to-day than formerly, but fairly comparable in its efficiency to the ballads, whether English or Homeric, to which he attaches a just importance. We refer to the English Bible and the English Liturgy, both of them books standing in the front rank of literature, and both full of object lessons as to what words mean, and as to what effects they can be so combined as to produce.

### The Late Sir Douglas Galton, K.C.B.

The death of Sir Douglas Galton at the comparatively early age, for the men of this generation, of 77 years, has removed one of the most conspicuous and most useful of modern sanitary reformers, and one to whom it was given, in no common measure, to witness the results of his labours. His attention seems first to have been directed to questions of public health by his appointment, soon after the close of the Crimean War, as a member of a commission which was instructed to visit and to report upon the barracks and military hospitals of Great Britain and Ireland, and afterwards upon those of the Mediterranean stations, for the purpose of endeavouring to apply to them, as far as circumstances would permit, the comparatively simple methods by which the late Sir Robert Rawlinson had brought about so signal an improvement in the health of the troops engaged in the campaign, to whom, prior to his reforms, disease had been almost precisely ten times as fatal as the weapons of the enemy. Captain Galton's observations during his service as a Commissioner led him to form very decided views with regard to the way in which hospitals should be constructed and administered; and, soon after the termination of this work, the Herbert Hospital at Woolwich was erected from his designs, and has abundantly justified

ANNOTATIONS—*continued.*

the great principles which he laid down. Before modern bacteriology had disclosed the chief reason for the value of cleanliness, Sir Douglas was unwearied in his advocacy of it on the basis of experience, and lost no opportunity, either at the British Association for the Advancement of Science, at the Social Science Congresses, or as a member of the Sanitary Institute, of impressing his own convictions upon the public. His sanitary work, however, constituted but a portion of his activity, which was extended to railway brakes, ventilation, submarine telegraphy, and a great variety of other subjects on which his training as an engineer qualified him to be an authority. Sir Douglas combined with his many accomplishments the modesty and the love of truth of a philosopher, as well as a remarkable capacity for seeing the humorous side of a discussion. He became of late years an habitual frequenter of the Athenæum Club, where, as well as in many other social circles, his genial smile and winning manners will long be missed by large numbers of his friends.

### Lady Henry Somerset's Home at Duxhurst.

The report of Lady Henry Somerset's Industrial Farm Colony for Inebriate Women is very interesting reading at the present time, when the prolonged and scientific treatment of inebriates has become a part of our national system of legislation. This colony has not been long started, and the cases dealt with in it are not numerous; but the success that has attended the work, though not complete, is very encouraging. Statistics are given of 112 cases, in 55 of which the treatment, tested by inquiry and examination after the patients had left the colony, has proved successful. A stay of one year under careful medical treatment, suitable work, and healthy environment is regarded as necessary to a cure; but several of the patients did not complete their term of treatment, and it would be more just to consider only those who give the treatment a proper chance; these numbered 83. When only these are reckoned, the proportion of cures is greater than if all who ever entered the colony, for however short a time, are included. Thus reckoned, it amounts to 67 per cent.; but, even including all the temporary inmates, the percentage is very nearly 50 per cent. Of the others 25 failed after the full year of treatment, 12 were removed before the period was up, 5 absconded, 3 proved incorrigible and had to be sent away, 6 were removed by the medical officer as unfit for treatment, 3 died, 2 were insane, and 1 remains doubtful. Taking the lowest estimate, half of these women, apparently hopeless drunkards, have been restored to decency, hope, and usefulness. It is to be noted, too, that 43 of the patients had inebriate family history, 8 came of families in which there was insanity, and 11 had ancestors who had died of paralysis. Thus, in the majority of cases, the family history showed tendencies which lead very easily to alcoholism. The inference seems to be that drunkenness, when an acquired habit, may with proper treatment be cured, but that the case becomes more difficult, though not hopeless, when the tendency is hereditary. The notorious Jane Cakebread was one of Lady Henry Somerset's failures; but at Duxhurst it was found out—and it is little to the credit of our dealings with habitual drunkards that it was not discovered before—that the poor creature was insane, and was fit only for an asylum. One of the advantages we may hope for from the establishment of reformatories for drunkards under Government control will be the earlier discovery of the real facts in such cases. Under the new *régime*, which we hope is about to dawn, it would not have been necessary for Jane Cakebread to go 300 times between the public-house and the police-court before the true nature of her ailment was discovered. It is only when the drunkard is under skilled supervision that it is possible to say when drunkenness is a moral defect which can be overcome, and when a disease beyond the patient's control. At Duxhurst the women are employed, not only in the ordinary domestic work connected with the homes, but in gardening and fruit and flower growing for market. The healthy outlet these occupations afford for their energies seems to be very useful in restoring the moral as well as the physical health which the patients have lost, giving them time to think but not time to mope. One word only remains to be said. The cases treated have been 112; the cases refused from lack of room and means to admit them have been 3,000. Three thousand women, weak and faulty, but not beyond redemption, who themselves or their friends are willing to pay for the year's treatment that promises them restoration—for the Duxhurst colony is not free, and cannot afford to take those who cannot contribute to their own support! And they have to be refused. No wonder Lady Henry Somerset asks for contributions to enable her to go on and increase her work.

### Mr. Rudyard Kipling.

The happy termination of the serious illness of "the American writer Kipling," as some egregious German journalists have lately described him, will be hailed with no common gratification wherever the English tongue is read or spoken. It has been the lot of few men to strike so boldly and so clearly the keynote of Imperial patriotism and of Imperial duty, or to do so much towards the reduction of "little Englandism" to its proper insignificance. Nor has his good influence ended here, since his illness, perhaps even more than his writings, has united the two great divisions of the Anglo-Saxon race by a tie of common sympathy which, however little it may be reducible to figures or be capable of being expressed in treaties, will be by no means without its effect upon social and political relations. It has been declared by humourists to be a well-known fact that Shakespeare was "an American citizen," and, although we shall not easily acquiesce in the German version of Mr. Kipling's nationality, we are more than ready to allow our transatlantic cousins to join with us in saying, "We are all proud of him." Nor will less sympathy be given to his noble wife, and to her heroic suppression of her grief at the loss of a daughter, lest its manifestation should diminish the once slender chances of her husband's recovery. The intelligence of this loss has now, we hear, been communicated to Mr. Kipling, and we join heartily in the universal hope that his recovery is sufficiently advanced to remove all anxieties about his future, although even this inevitable sorrow mingles with the thankfulness which he cannot fail to experience. Whatever it may be to others, life must surely be sweet to one who is so gifted with the power of using it to advantage.

